# Poria Acid, Triterpenoids Extracted from *Poria cocos*, Inhibits the Invasion and Metastasis of Gastric Cancer Cells

**DOI:** 10.3390/molecules27113629

**Published:** 2022-06-06

**Authors:** Haibo Wang, Yuanyuan Luo, Zewen Chu, Tengyang Ni, Shiya Ou, Xiaojun Dai, Xiaochun Zhang, Yanqing Liu

**Affiliations:** 1 Institute of Translational Medicine, Medical College, Yangzhou University, Yangzhou 225001, China; lyy528539840@163.com (Y.L.); 17521176065@163.com (Z.C.); sfntylzyssyzu@163.com (T.N.); osywhb@163.com (S.O.); 2 The Key Laboratory of Syndrome Differentiation and Treatment of Gastric Cancer of the State Administration of Traditional Chinese Medicine, Yangzhou 225001, China; dxj2319@163.com; 3 Yangzhou Hospital of Traditional Chinese Medicine, Yangzhou 225001, China

**Keywords:** *Poria cocos*, poria acid, anti-gastric cancer, invasion and metastasis

## Abstract

Background: *Poria cocos (P. cocos)* is an important medicinal fungus in traditional Chinese medicine. Poria acid (PA), a triterpenoid compound, is an effective component of traditional Chinese medicine *P. cocos*. This experiment investigated the anti-gastric cancer biological activity of PA in vitro. Methods: The effect of PA on the viability of gastric cancer cells was detected by the thiazolyl blue (MTT) assay. Cell adhesion assays were used to detect changes in the adhesion of cells treated after PA (0, 20, 40, and 80 µmol/L). The ability of cell invasion and migration were detected by Transwell assays and wound healing assays. A high-content imaging system was used to dynamically record the motility of the gastric cancer cells after PA (0, 20, 40, and 80 µmol/L) treatment. Western blotting was used to detect the expression of epithelial–mesenchymal transformation (EMT), invasion and migration related proteins. Results: The MTT assay showed that the proliferation of gastric cancer cells was significantly inhibited after PA treatment. Cell adhesion experiments showed that the adhesion of gastric cancer cells was significantly decreased after PA treatment. Compared with the control group, the wound healing area of the gastric cancer cells treated with different concentrations of PA decreased. The Transwell assay showed that the number of gastric cancer cells passing through the cell membrane were significantly reduced after PA treatment. In addition, after PA treatment, the cells’ movement distance and average movement speed were significantly lower than those of the control group. Finally, PA can significantly alter the expression of EMT-related proteins E-cadherin, N-cadherin, and Vimentin and decreased the expressions of metastasis-related proteins matrix metalloproteinase (MMP) 2, MMP-9 and tissue inhibition of matrix metalloproteinase (TIMP)1 in the gastric cancer cells. Conclusions: Triterpenoids from *P. cocos* have significant biological activity against gastric cancer, and the mechanism may be involved in the process of epithelial–mesenchymal transformation.

## 1. Introduction

Gastric cancer (GC) is the fifth most common malignancy worldwide, with a high mortality rate and is the third leading cause of cancer death [1]. Some scholars have reported that the incidence of GC is higher in men than in women, even two to three times higher in women, there are geographical differences, and the incidence is higher in developing countries [2,3]. The most obvious feature of GC is its easy invasion and metastasis, which is also the main cause of death in GC patients [4]. Currently, the only possible cure for GC is surgical resection. However, due to atypical symptoms, most GC patients are already in the middle and late stages when they are diagnosed, so some patients still have poor postoperative recovery [5,6]. Therefore, the inhibition of GC cell metastasis is particularly important in tumor therapy including the inhibition of GC cell migration and the invasion and inhibition of GC cell adhesion.

Tumor metastasis is a complex multi-step process involving multiple genes and their product, and EMT is related to the occurrence, invasion, and metastasis of tumors [7,8]. Epithelial–mesenchymal transformation is a process in which epithelial cells lose polarity and intercellular adhesion and transform into mesenchymal cells under normal or specific pathological conditions [9]. In the progression of malignancy, tumor cells hijack this process to change their cell morphology, thus increasing their invasiveness and metastasis ability [10]. Many studies have shown that EMT is closely related to the invasion and metastasis of GC [11,12]. Therefore, EMT intervention to inhibit the invasion and metastasis of GC has become the focus of many scholars [13,14,15,16]. In addition, matrix metalloproteinases (MMPs) can degrade various proteins in the extracellular matrix (ECM) and play a key role in the process of EMT [17]. Therefore, research on therapeutic strategies and drug development targeting EMT and MMPs has become a research hotspot. More and more people are turning their attention to the anti-tumor aspect of traditional Chinese medicine (TCM), because of its monomer good curative effect, and less side effects in clinical application can improve the patients’ survival rate and survival quality. At present, TCM has outstanding efficacy in the prevention and treatment of the invasion and metastasis of gastric cancer, breast cancer, lung cancer, liver cancer, colorectal cancer, and other cancers [18]. As a commonly used medicine, *P. cocos* has a very long history of use in traditional Chinese medicine. *P. cocos*, polyporaceae, is the dried sclerotia of pseudoporus fungus poria, often parasitic on the roots of pine trees, shaped like sweet potatoes, spherical, light-brown or dark-brown outer skin, and a pink or white interior. *P. cocos* is mainly produced in Anhui, Jiangxi, Jiangsu, Zhejiang, and other places in China. It has the effect of infiltrating dampness and diuretic water, benefiting the spleen and stomach, calming the heart, and soothing the mind. Recent studies have also found that *P. cocos* has an obvious anti-tumor effect. Studies have found that *P. cocos* can inhibit the proliferation and apoptosis of cancer cells [19]. Other studies have revealed that *P. cocos* inhibits the invasion of ovarian cancer cells by participating in the E-cadherin/β-catenin signaling pathway [20], and inhibits the invasion of pancreatic cancer cells by reducing the expression of MMP-7 [21,22]. However, the molecular mechanism of the anti-tumor effect of *P. cocos* is still unclear. The most important thing is that among the active ingredients of *P. cocos*, the specific anti-tumor active ingredient needs to be determined by researchers urgently. There are many chemical components in *P. cocos* including polysaccharides and triterpenoids, sterols, volatile oils, and proteins [23]. Poria acid (PA) is a single white powder with the molecular formula C_33_H_52_O_5_ and molecular weight of 528.763. PA is an important representative of triterpenoids of *P. cocos* and is one of the effective components of *P. cocos* that is of wide concern. This study investigated the effect of PA on GC proliferation, and clarified the effect and underlying molecular mechanism of PA in inhibiting GC metastasis in vitro.

## 2. Materials and Methods

### 2.1. Drugs

Poria acid (standard substance, HPLC 97%) was purchased from Shanghai Yuanye Technology Co. Ltd., Shanghai, China (Cat.no. B20400).

### 2.2. Reagents

RPMI medium modified with 2.05 mM L-glutamine (HyClone, Waltham, MA, USA, Cat. no. SH30809.01); fetal bovine serum (Gibco, Waltham, MA, USA, Cat. no. 10099141); Transwell (Corning, New York, NY, USA, Cat. no. 356234); 3-(4,5-Dimethylthiazol-2-yl)-2,5-diphenyltetrazolium bromide (MTT, Merck, Berlin, Germany, Cat. no. M5655); MMP-2, MMP-9, E-cadherin, N-cadherin, Vimentin, and β-actin (Thermo Fisher Scientific, Waltham, MA, USA, Cat. no. PA5-85197, PA5-16509, PA5-32178, PA5-29570, MA5-16409, PA146296); TIMP-1 (Abcam, Cambridge, UK, Cat. no. ab211926).

### 2.3. Cell Culture

The AGS and MKN-28 human gastric cancer cell lines were purchased from China Procell Life Science & Technology Co. Ltd., Wuhan, China; The AGS and MKN-28 cells were cultured in RPMI-1640 medium containing 10% fetal bovine serum in an incubator at 37 °C and 5% CO_2_.

### 2.4. Cell Viability Assay

The GC cells were digested by trypsin and re-suspended into a single cell suspension. Cells were seeded in 96-well plates with 4 × 10^3^ cells per well and were cultured in an incubator at 37 °C. After being treated with different concentrations of PA for 24 h and 48 h, the GC cells were incubated with the added MTT for another 4 h. After the supernatant was removed, 100 μL of dimethyl sulfoxide (DMSO) was added to each well. The 96-well plate was placed in the automatic microplate analyzer and shaken for 10 min to fully dissolve the crystals. The absorbance (A) value of each well was measured at 490 nm. The inhibition rate (%) was calculated as [1 − (cell A in the drug group/cell A in the control group)] × 100%.

### 2.5. Cell Adhesion Assay

The GC cells were seeded in 6-well plates and intervened with PA for 24 h. The Matrigel gel was diluted 8-fold with serum-free medium, and the diluted Matrigel gel was added to a 24-well plate in a volume of 300 μL. After drying and solidification, the unsolidified glue was washed with phosphate buffer saline (PBS). The cells were seeded into 24-well plates at 20,000 cells per well, and incubated in a 37 °C incubator for 90 min. Unadherent cells were gently washed with PBS and adherent cells were fixed with methanol for 30 min. The fixed cells were photographed and counted with an inverted microscope after they were stained with crystal violet for 10 min and washed with water.

### 2.6. Wound Healing Assay

The GC cells were trypsinized and seeded in 6-well plates at 3 × 10^5^ per well. When the degree of cell fusion was greater than 80%, draw lines were drawn on the monolith of the fused cells using the tip of a 200 µL pipetting tube. Next, the cells were treated with PA with final concentrations of 0, 20, 40, and 80 µmol/L. The 96-well plates were cultured in an incubator at 37 °C. Pictures were taken at 0 h, 24 h, and 48 h, respectively, with an inverted microscope. Wound healing degree (%) was calculated as ((scratch width of control group − drug group)/scratch width of control group)) × 100%. The scratch width was measured by ImageJ software.

### 2.7. Transwell Chamber Assay

Cell migration assays were performed using 24-well Transwell chambers with 8.0 µm pore size polycarbonate membranes. GC cells were digested and suspended in serum-free medium, and inoculated in the upper chamber at 2 × 10^5^ per well. Different concentrations of PA were added into the lower chamber and the cells were continuously cultured for 24 h. The cells were fixed with methanol for 30 min and stained with crystal violet for 15 min. The uncrossed cells on the membrane surface at the bottom of the upper chamber were gently wiped with cotton swabs. Ten fields were randomly selected under an inverted microscope. For the invasion assay, Matrigel was diluted 1:8 in serum-free medium, and added to the Transwell upper chamber. The remaining steps were the same as the migration assay. The number of transmembrane cells was calculated using ImageJ software.

### 2.8. High-Content Imaging Technology

The GC cells were digested and inoculated into 96-well plates at a density of 4 × 10^3^ cells per well. After cell adherence, the cells were treated with PA, and incubated for 12 h in an incubator at 37 °C. The board was placed in a PerkinElmer Operetta CLS high content Imaging System machine for further incubation for 12 h, and the Harmony 4.1 software was used for data collection and analysis.

### 2.9. Western Blot Analysis

The GC cells were inoculated into 6-well plates and treated with PA at the final concentrations of 0, 20, 40, and 80 µmol/L for 24 h. The total protein of each group was extracted. The protein lysates were separated in a 10% SDS-PAGE gel and transferred to polyvinylidene fluoride (PVDF) membranes. After being blocked with 5% skim milk for 2 h, the membrane was incubated with the primary antibody at 4 °C for 12 h, and then incubated with the secondary antibody at room temperature for another 2 h. The protein bands were detected by a gel imaging analysis system.

### 2.10. Statistical Analysis

All data were averaged from at least three independent trials. The data within the group conformed to a normal distribution. Graph Prism 8.0 software (GraphPad Software, Inc., San Diego, CA, USA) was used for ordinary one-way ANOVA to statistically significant differences. The data are shown as the means ± standard deviations. * *p* < 0.05, ** *p* < 0.01, *** *p* < 0.001, **** *p* < 0.0001 was considered statistically significant.

## 3. Results

### 3.1. PA Inhibits the Viability of GC Cells

**Figure 1:**
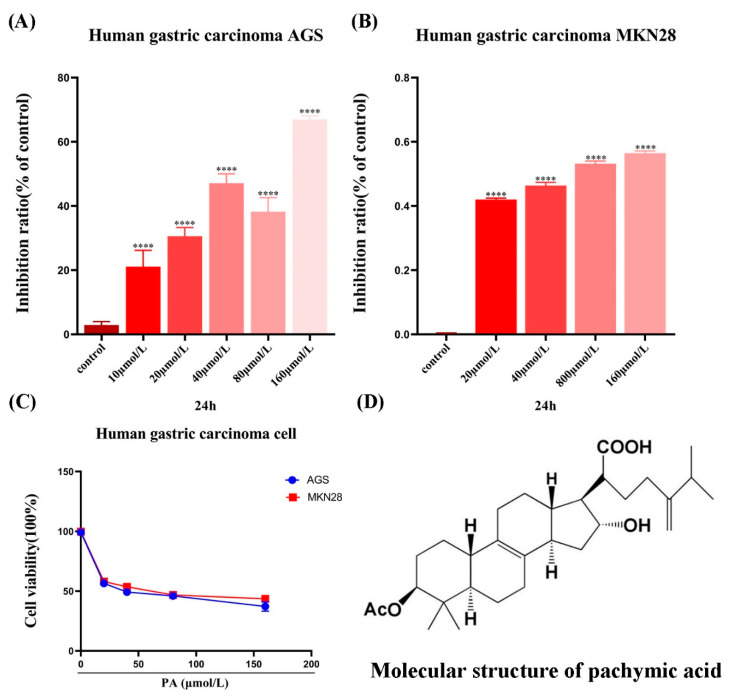
The effect of PA on the GC cell viability. (**A**,**B**) Different concentrations (0, 10, 20, 40, 80, 160 μmol/L) of PA inhibited the growth of GC cells for 24 h treatments. (**C**) The viability of the GC cells treated with PA for 24 h. (**D**) Chemical structure of Poria acid. **** *p* < 0.0001.

The results showed that compared to the control group, PA had a certain anti-proliferation effect at different concentrations. In general, the effect of PA on the GC cells was time-dependent and concentration-dependent (Figure 1A–C). In order to exclude the cytotoxicity of PA, only low-concentrations of PA (0, 20, 40, and 80 µmol/L) were selected in the subsequent experiments to further investigate the effects of PA on tumor invasion and migration.

### 3.2. PA Reduces the Adhesion of GC Cells

**Figure 2:**
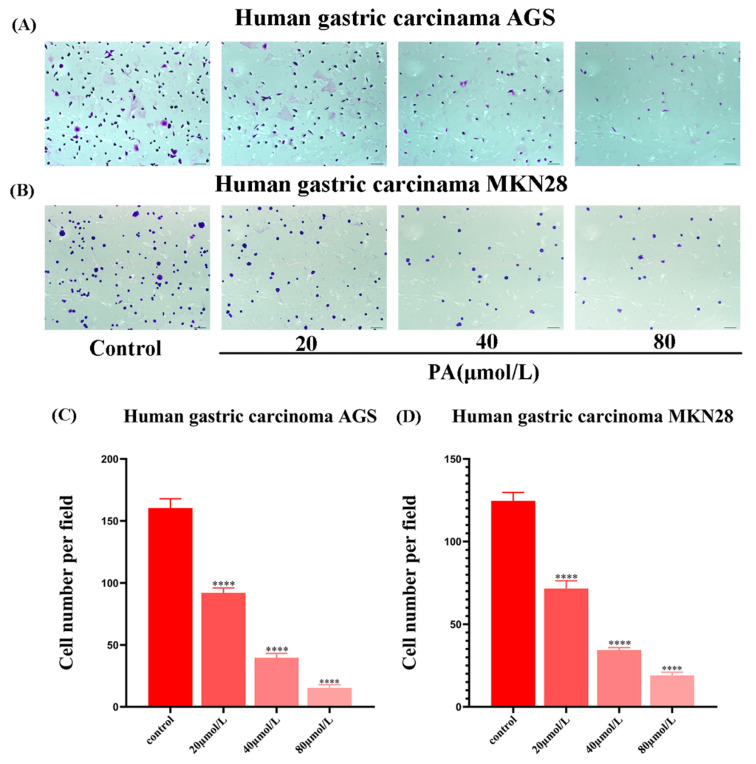
The effect of PA on the GC cell adhesion. (**A**,**B**) Photos of the GC cell adhesion after crystal violet staining. (**C**,**D**) Different concentrations (0, 20, 40, 80 μmol/L) of PA inhibited the adhesion of the GC cells. **** *p* < 0.0001.

The number of adherent cells was significantly reduced after PA treatment of the GC cells for 24 h (Figure 2A). After further statistical analysis, the results showed that compared to the control group, the adhesion of GC cells after PA treatment was significantly reduced, and the difference was significantly different (Figure 2).

### 3.3. PA Inhibits the Migration of GC Cells

**Figure 3:**
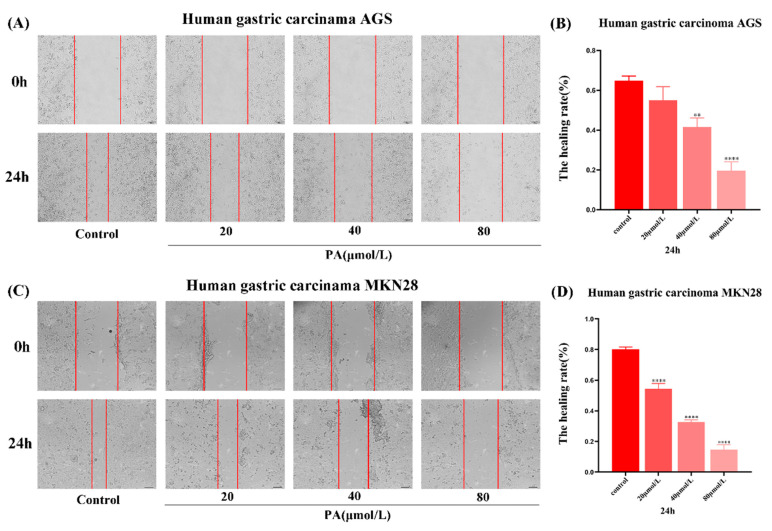
The effect of PA on the GC cell migration. (**A**,**C**) Photos of the GC cell migration distance after being treated with PA at different concentrations (0, 20, 40, 80 μmol/L) for 24 h and 48 h. (**B**,**D**) Statistical graph of the wound healing rate. The cells were imaged under a microscope at 200× magnification. All scales are 200 µm. ** *p* < 0.01, **** *p* < 0.0001.

The wound area of the GC cells treated with PA was significantly larger than that of the control group. The healing ability of the PA treated GC cells was significantly weakened in a concentration-dependent manner (Figure 3). These results indicate that PA could inhibit the migration of the GC cells. As the time dependence is not significant, the effect of 24 h PA treatment on the invasion and migration of GC cells will be observed in the following experiments.

### 3.4. PA Inhibits GC Cell Invasion and Migration

**Figure 4:**
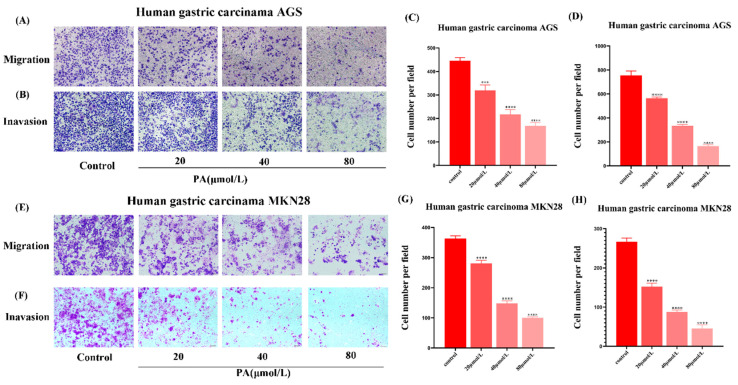
The effect of PA on the invasion and metastasis of the GC cells. (**A**,**B**,**E**,**F**) Transwell chamber photograph of the invasion and metastasis assay of the GC cells after different concentrations (0, 20, 40, 80 μmol/L) of PA treatment for 24 h. (**C**,**D**,**G**,**H**) The summary of the data for the Transwell migration and invasion assays. The GC cells were imaged under a 200× microscope. All scales are 200 µm. *** *p* < 0.001, **** *p* < 0.0001.

Compared with the control group, the migration of the GC cells were significantly reduced after 24 h PA treatment (Figure 4A,E). The invasion assay results showed that the invasion ability of the GC cells was also inhibited by PA in a concentration-dependent manner. With the concentration in PA increasing, the invasion ability of the GC cells decreased (Figure 4B,F). The above data together suggest that PA can effectively inhibit the invasion and migration of GC cells.

### 3.5. PA Inhibited the Dynamic Migration of GC Cells

**Figure 5:**
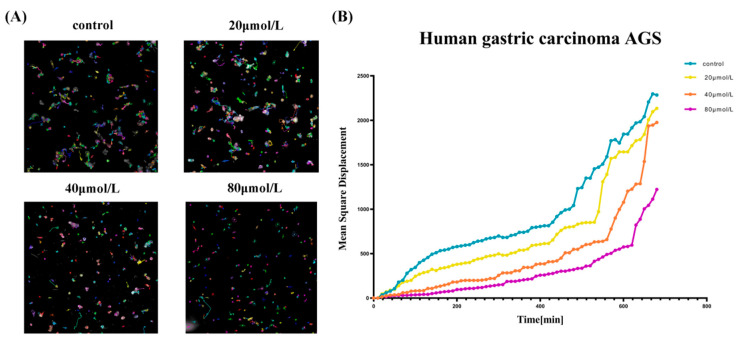
The effect of PA on the dynamic migration of the AGS cells. (**A**) The high-intension imaging system tracks the cell’s dynamic processes for 12 h. (**B**) The mean square displacement was plotted against observation time.

The high-content imaging results indicated that AGS cells exhibit different degrees of motility inhibition with increasing PA concentration. The mean azimuth shift diagram and cell displacement diagram were drawn (Figure 5). It can be concluded that with the increase in the drug concentration, the migration trajectory of cells became shorter and the migration ability was weakened.

### 3.6. PA Inhibits the Movement Ability of AGS Cells

**Figure 6:**
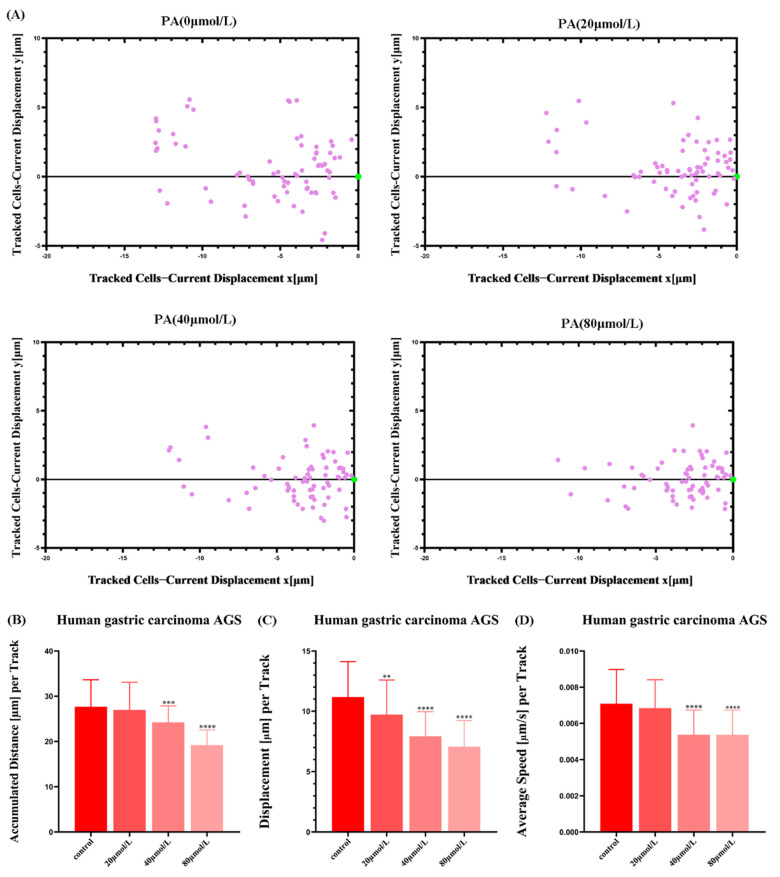
The effects of PA on the movement distance and speed of the AGS cells. (**A**) Each point corresponds to the displacement of a cell at a given time point. (**B**) Accumulated distance (µm) per track. (**C**) Displacement (µm) per track. (**D**) Average speed (µm) per track. ** *p* < 0.01, *** *p* < 0.001, **** *p* < 0.0001.

In order to observe the positions of the GC cell populations at different time points in real-time, the movement trajectories of the cell populations were drawn based on the high-content data. The results showed a narrower distribution of the trajectories of the GC cell populations after PA treatment compared to that of the control group (Figure 6). This again shows that PA can reduce the motility of the GC cells.

### 3.7. PA Affects the Expression of EMT-Related Proteins and MMP-Related Proteins in AGS Cells

**Figure 7:**
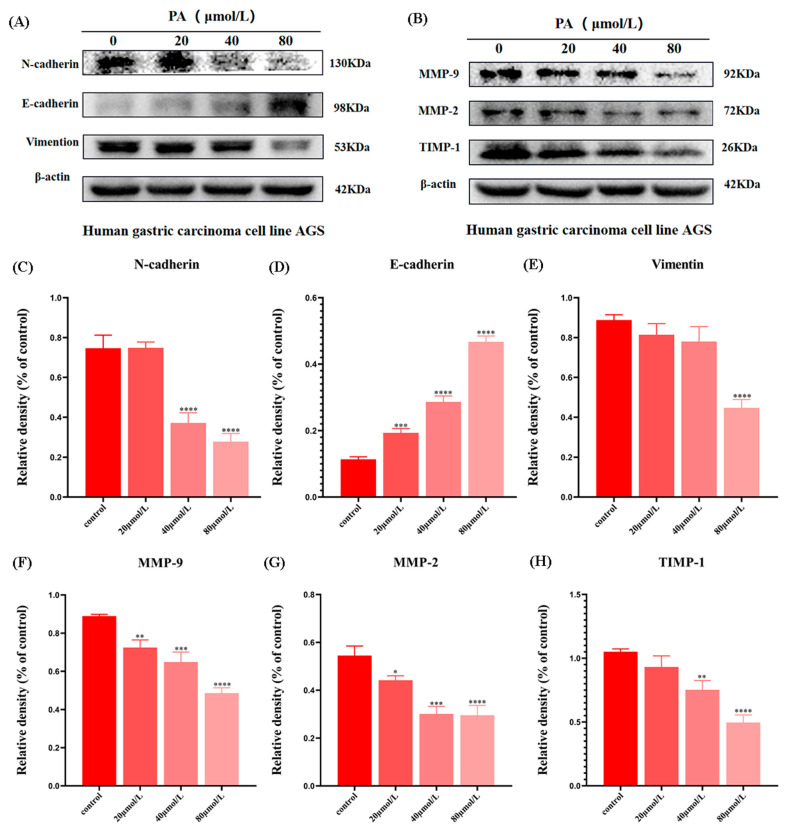
The effects of PA on the expression of EMT and MMPs. (**A**,**C**–**E**) The Western blot band and quantification relative statistics of EMT-related proteins in the AGS cells. (**B**,**F**–**H**) The Western blot band and quantification relative statistics of the metastasis-associated proteins in AGS cells. * *p* < 0.05, ** *p* < 0.01, *** *p* < 0.001, **** *p* < 0.0001.

The changes in these proteins can directly reflect the invasion and metastasis ability of the GC cells. The Western blot results showed that compared with the control group, the expression of epithelial marker E-cadherin was significantly increased, while the expression of mesenchymal marker Vimentin and N-cadherin was decreased (Figure 7A and C–E). In addition, it was further found that PA also inhibited the expression of MMP-related proteins (Figure 7B and F–H). These results suggest that PA can inhibit the invasion and migration of AGS cells, possibly by inhibiting the expression of EMT and MMPs.

## 4. Discussion

The invasion and metastasis of GC is the fundamental cause of GC treatment failure and the main cause of death in GC patients [24]. The main treatment for GC metastasis is palliative treatment based on chemotherapy [25]. However, due to the existence of chemotherapy drug resistance immunosuppression and poor physical conditions, patients often cannot tolerate multiple chemotherapy. TCM has obvious advantages in the prevention and treatment of GC invasion and metastasis. Many TCM monomers and TCM compounds have significant effects on the intervention of GC invasion and metastasis [26,27,28]. As a traditional Chinese medicine, *P. cocos* can regulate gastrointestinal function and protect the liver. Recent pharmacological studies on *P. cocos* have found that *P. cocos* also has significant anti-tumor effects [19,20]. As the main component of *P. cocos* triterpenes, PA is likely to be an important anti-tumor component in *P. cocos*. In this study, the effect of PA on inhibiting the invasion and metastasis of GC cells in vitro was reported. This experiment first examined the effect of PA on the GC cell proliferation. The proliferation of tumor cells was the basis of tumor invasion and metastasis [29]. This study showed that PA inhibited the proliferation of GC cells in a concentration-dependent manner. This lays the foundation for further exploration of the effect of PA on the invasion and metastasis of GC cells. The movement of cancer cells is a continuous process. The inhibition of cell movement may be an effective way to suppress tumor metastasis. Cell adhesion is one of the key steps in cell motility and is a necessary condition for GC cell directional movement. Inhibiting the adhesion of GC cells can effectively inhibit the invasion and metastasis of GC cells. The results of the cell adhesion experiments showed that PA could significantly reduce the adhesion of GC cells. This indicates that PA can inhibit the cell movement at the initiation of cell movement. To further confirm the effect of PA on the invasion and metastasis of GC cells, the wound healing assay and Transwell assay were performed. These results suggest that PA can significantly inhibit the invasion and metastasis of GC cells. These further indicate that PA could inhibit the invasion and metastasis of GC cells. PA may be one of the important antitumor components of *P. cocos*. In order to intuitively and accurately study the effect of PA on GC cell metastasis, a high-content cell real-time dynamic tracking system was used to record the movement state of the GC cells treated with PA. The cell dynamic tracking reconfirmed the results of the above experiments. Both in terms of the instantaneous velocity and the average displacement, the GC cells treated with PA were smaller than those of the control group. The above experiments allowed us to intuitively observe the effect and process of PA inhibiting the invasion and metastasis of GC cells. To further elucidate the molecular mechanism of the PA inhibition of GC cells, the changes of PA on the EMT and metastasis-related proteins of GC cells were examined.

Epithelial–mesenchymal transformation refers to the transformation of epithelial cells into mesenchymal cells under the stimulation of some factors, which is critical for tumor cells to acquire the ability of invasion and metastasis [30,31]. E-cadherin is considered to be a key factor that maintains the stability and cell polarity between the epithelial cells [32]. Once the expression of E-cadherin is decreased, the adhesion between the epithelial cells is directly decreased, which promotes the invasion of epithelial tumor cells [33]. On the other hand, the acquisition of interstitial characteristics is characterized by the fusiform interstitial morphology of cells, accompanied by the increase in the interstitial markers N-cadherin and Vimentin [34]. Specifically, the downregulation of E-cadherin could be balanced by the increased expression of N-cadherin [35,36]. Vimentin, as a typical marker, can regulate a variety of cell types involved in cell migration and enhance cell invasiveness [37,38]. This study showed that PA increased the expression of E-cadherin and decreased the expression of N-cadherin and Vimentin. These results suggest that PA may inhibit the invasion and metastasis of GC cells by inhibiting the EMT process.

The extracellular matrix is an extracellular network that supports and immobilizes cells and plays an important role in tumor cell metastasis [39,40]. MMPs play an important role in the degradation of the extracellular matrix and basement membrane [41]. MMP-2 and MMP-9 can degrade the ECM, greatly change the viscosity and mobility of the tumor, and promote invasion and metastasis [42,43]. This study confirmed the inhibitory effect of PA on MMPs. These findings suggest that the inhibition of GC cell invasion and metastasis by PA may be related to the inhibition of the MMP protein expression.

In conclusion, this study found that a triterpenoid in *P. cocos* (PA) could inhibit the invasion and metastasis of GC cells. Many results also further confirmed that PA could inhibit the EMT process and MMP protein expression in GC cells. These data provide new evidence for understanding the anticancer mechanism of *P. cocos* in vitro. This study showed that PA, as the active ingredient in *P. cocos*, is likely to be the key anti-tumor ingredient in *P. cocos*. This will lay a great experimental foundation for the further development and use of *P. cocos* as an anticancer drug.

## Data Availability

All data included in this study are available upon request by contact with the corresponding author.

## Sample Availability

Samples of the compounds are available from the authors.
